# Beyond Mutations: Additional Mechanisms and Implications of SWI/SNF Complex Inactivation

**DOI:** 10.3389/fonc.2014.00372

**Published:** 2015-02-27

**Authors:** Stefanie B. Marquez, Kenneth W. Thompson, Li Lu, David Reisman

**Affiliations:** ^1^Department of Medicine, Division of Hematology/Oncology, University of Florida, Gainesville, FL, USA; ^2^Department of Pathology, University of Florida, Gainesville, FL, USA

**Keywords:** Brahma, chromatin remodeling, epigenetic BRG1, SMARCA2, SMARCA4

## Abstract

SWI/SNF is a major regulator of gene expression. Its role is to facilitate the shifting and exposure of DNA segments within the promoter and other key domains to transcription factors and other essential cellular proteins. This complex interacts with a wide range of proteins and does not function within a single, specific pathway; thus, it is involved in a multitude of cellular processes, including DNA repair, differentiation, development, cell adhesion, and growth control. Given SWI/SNF’s prominent role in these processes, many of which are important for blocking cancer development, it is not surprising that the SWI/SNF complex is targeted during cancer initiation and progression both by mutations and by non-mutational mechanisms. Currently, the understanding of the types of alterations, their frequency, and their impact on the SWI/SNF subunits is an area of intense research that has been bolstered by a recent cadre of NextGen sequencing studies. These studies have revealed mutations in SWI/SNF subunits, indicating that this complex is thus important for cancer development. The purpose of this review is to put into perspective the role of mutations versus other mechanisms in the silencing of SWI/SNF subunits, in particular, BRG1 and BRM. In addition, this review explores the recent development of synthetic lethality and how it applies to this complex, as well as how BRM polymorphisms are becoming recognized as potential clinical biomarkers for cancer risk. *Significance*: Recent reviews have detailed the occurrence of mutations in nearly all SWI/SNF subunits, which indicates that this complex is an important target for cancer. However, when the frequency of mutations in a given tumor type is compared to the frequency of subunit loss, it becomes clear that other non-mutational mechanisms must play a role in the inactivation of SWI/SNF subunits. Such data indicate that epigenetic mechanisms that are known to regulate BRM may also be involved in the loss of expression of other SWI/SNF subunits. This is important since epigenetically silenced genes are inducible, and thus, the reversal of the silencing of these non-mutationally suppressed subunits may be a viable mode of targeted therapy.

## Introduction

SWI/SNF is a chromatin-remodeling complex first described in the early 1990s. These studies showed that this complex opens up the chromatin by shifting the position of histones and making the DNA accessible to transcription factors and key cellular proteins. In yeast, there is only a single complex, but in mammals there are at least three different complexes. Each complex is composed of one of two ATPase subunits, Brahma (BRM/SMARCA2) or Brahma related gene 1 (BRG1/SMARCA4) and 8–10 other subunits that results in a 2-MDa complex. In yeast, this complex regulates about 5–6% of the genome, while in mammalian organisms, it is not known how many genes are regulated by this complex and how many protein interactions occur in conjunction with this complex. However, SWI/SNF does not interact with one type of transcription factor, but rather, it regulates the function of many diverse genes as well as the function of many signaling pathways. SWI/SNF has been found to function in DNA repair, growth control, differentiation, development, cellular adhesion, and immunity. In most cases, SWI/SNF functions in general to maintain homeostasis and normal cellular function. The function and composition of SWI/SNF is detailed in many other reviews and is not the focus of this review. As SWI/SNF subunits are frequently found to be mutated in different tumors, we compared published mutation data (COSMIC and Atlas databases) with published IHC (when available) to determine how frequently mutations underlie the loss of expression of different SWI/SNF units. As detailed below, in many cases, mutations cannot account for why certain SWI/SNF subunits are silenced in cancer, which suggests that other mechanisms likely underlie the loss of these proteins in cancer.

## Differing Roles for SWI/SNF in Cancer, Depending on the Context

SWI/SNF functions as an epigenetic regulator that opens and closes the chromatin, thereby helping to turn gene expression on and off. In this capacity, the complex serves as both an oncogene and a tumor suppressor, depending on the protein with which it is interacting (1). Abrogation of SWI/SNF can impact the normal functions of DNA repair, cellular differentiation, development, and cell adhesion (2). Its diversity in function has been recently demonstrated by contrasting sets of publications about the role of one of its two catalytic subunits, Brahma (BRM, or SMARCA2), in cancer. Traditionally, BRM has been viewed as a tumor susceptibility or tumor suppressor protein because its loss can potentiate the development of tumors in murine cancer models (3, 4) as well as portend worse clinical survival in lung cancer patients (5, 6). In addition, BRM loss in non-transformed cell lines has been shown to prevent cells from entering a canonical quiescent state (7), which further attests to the growth-inhibitory functions ascribed to BRM. Several labs have shown that BRM is necessary for RB function (8–10): it is known to bind to RB (11), and together, RB and BRM suppress the activity of E2F (12). A constitutively active form of RB fails to inhibit growth when introduced into BRM-deficient cell lines, as well as in cells that are deficient in its homolog, Brahma-related gene 1 (BRG1, or SMARCA4), which is the second catalytic subunit of SWI/SNF (10). In fact, re-expression of BRM can inhibit growth and induces differentiation in a variety of BRG1/BRM-deficient cell lines (8), a process that is disrupted by viral proteins that bind to RB, such as E1A or E7 (8, 13). Moreover, BRM re-expression has been shown to antagonize KRAS-transformed cells by partially reversing the transformed phenotype and substantially slowing growth; based on these data, these authors concluded that increased BRM expression promotes the entry of cells into G1/G0, while the loss of BRM expression may foster oncogenic transformation (14). BRM has been shown to promote exit from the cell cycle and cell differentiation in retinal ganglion cells (15). Re-activation of BRM and SWI/SNF appear to foster the active, hypophosphorylated form of RB through the inhibition of cyclin D1 by directly binding to its promoter and stimulating the downregulation of cyclin D1 as well as the induction of p16 (16, 17). Thus, numerous studies have clearly defined a growth-inhibitory role for BRM and SWI/SNF.

Apparently contrasting sets of observations, however, suggest a dichotomous function for BRM. These findings imply that BRM and SWI/SNF may not solely function in growth-inhibitory pathways but may also serve in a net stimulatory capacity. Thus, the function of BRM depends on the context. This concept has been directly observed in BRG1-deficient cell lines where the knockdown of BRM actually slows growth. This has been described as synthetic lethality – a term, which describes the situation where the loss of function of two or more genes leads to cell death, but where the loss of function in only one of the genes is not lethal. This finding of synthetic lethality after BRM knockdown has also been found to occur in xenograft models (18–20). These data indicate that BRM is necessary for stimulatory pathways – or, alternatively, that BRM may act as an antagonist to growth-inhibitory pathways (i.e., it acts as a dominant negative). Potentially important to these observations is the fact that BRM has been shown to be regulated post-translationally, where the acetylation of BRM blocks BRM-dependent gene expression (16) and conversely, the removal of BRM acetylation causes growth arrest (16). In at least a subset of the cell lines used to demonstrate synthetic lethality, BRM is actually known to be acetylated, which suggests that its growth-inhibitory functions are likely turned off (unpublished observations) (16, 21).

In this particular context, which in this case is in BRG1-deficient genotypes, experimental silencing of BRM has been observed to significantly slow cancer cell growth rather than promote it (19). At least two rationales might explain this observed phenomenon. First, if BRM and SWI/SNF function in the growth-promoting pathway, then their suppression would diminish their respective contributions to this pathway and would likely cause slower growth, as observed. To this end, SWI/SNF and BRM have been shown to be cofactors for such oncogenes as C-Jun and C-Myc (22, 23). Second, at least a subset of the cells that have demonstrated synthetic lethality harbor a form of BRM that is acetylated at the C-terminus (16), which means that BRM is likely inactivated in these cell lines (21). The frequency of BRM acetylation in primary tumors is not yet known, but BRM acetylation is estimated to occur in 65–70% of cancer cell lines, which suggests that BRM acetylation also occurs frequently in primary tumors (21). We have shown that the deacetylation of BRM promptly induces growth arrest (21). In this capacity, BRM may be functioning as a dominant negative, much like p53 does when mutated. Alternatively, the role of BRM may be dictated by its acetylation state, where its unacetylated form binds to RB and RB2 and enhances growth inhibition, and its acetylated form antagonizes RB and also functions in growth pathways such as those driven by oncogenes (e.g., C-Myc and C-Jun). We favor the latter hypothesis, as it explains the majority of experimental observations and minimizes the apparent contradictions in the data, for example, where BRM re-expression as well as BRM knockout can both result in growth inhibition in certain cellular contexts, as outlined in our recent Cancer Research editorial (24). In addition, there are at least three different acetylation sites. Whether each acetylation site governs a different activity of BRM or all three are required to inactivate BRM is not yet known. Nevertheless, these new data demonstrate that BRM, BRG1, and SWI/SNF have potentially different roles depending on the context.

## Synthetic Lethality and the Clinical Silencing of *BRM*

Based on the observation that the knockdown of BRM in BRG1-deficient cell lines induces synthetic lethality, the clinical silencing of BRM has been suggested as a therapeutic strategy. Yet, this approach may not take into account the other anti-cancer functions of BRM and SWI/SNF. SWI/SNF and BRM are known to regulate cellular adhesion, DNA repair, differentiation, and expression of MHC class 1 and 2 proteins (2). Hence, while their suppression in certain circumstances might promote growth inhibition, it is equally possible such a strategy could have adverse clinical consequences by accelerating de-differentiation and promoting metastasis by inhibiting the expression of adhesion proteins such as E-cadherin, CEACAM1, integrins, and CD44, which are known to be regulated by SWI/SNF (2, 25). Moreover, the loss of both BRG1 and BRM would favor the inhibition of DNA repair (2, 25), and in particular, such key DNA repair genes as GADD45 (26), which in turn could enhance tumor progression. As SWI/SNF is reported to regulate MHC class 1 and 2 proteins (27–29), the silencing of BRM might result in the loss of these proteins, impairing the normal function of the immune system (30). BRM and the SWI/SNF complex have roles in the maintenance of genomic integrity and are required for the correct segregation of chromatids (7). BRM loss might also impact gene expression by changing the splicing pattern of genes such as CD44, Cyclin D1, and BIM (31). There are many positive functions governed by SWI/SNF that are yet to be well defined, and they might also be compromised if BRM is clinically silenced.

It might be a better approach to understand the mechanism that underlies synthetic lethality in this context before pursuing it as a mode of clinical therapy. For example, if BRM acetylation is truly a component of this phenomenon, the reversal of acetylation might be a prudent and feasible approach to affect the growth control function of BRM as well as the restoration of BRM’s other anti-cancer functions. This is because deacetylation *in vitro* results in growth inhibition and in the restoration of the transcriptional ability of BRM (16, 21). To this end, we know that BRM acetylation is governed by the functional balance of at least three proteins: HDAC2, KAT2B, and KAT8 (21). It is possible that aberrant KAT activity could be driving the acetylation of BRM in cancer, as KATs are known in certain cancer types to be hyperactive due to overexpression and the formation of hybrid/fusion proteins (32–34). In particular, as synthetic lethality has been observed in lung cancer-derived cell lines, it is interesting to note that KAT2B (GCN5, PCAF) is overexpressed in lung cancer – and this same protein has been shown to drive the expression of the cell cycle genes *E2F1*, *cyclin D1*, and *cyclin E1* (35). The targeting and inhibition of KAT2B also inhibit cancer by a variety of mechanisms (36–38), besides restoring BRM function. Hence, it may be more clinically appropriate to restore BRM function and its ability to inhibit growth by simply targeting KAT2B activity, rather than to silence BRM and risk the loss of BRM’s other anti-cancer functions. Further studies into the mechanism of BRM synthetic lethality and acetylation are needed to complete this complex picture before the best clinical avenues can be pursued.

## The RB Pathway and SWI/SNF

The SWI/SNF complex has been implicated in the function of the RB pathway ever since it was identified in mammalian cells. In two papers published in the mid-1990s (8, 39), Dr. Goff’s research group was one of the first to show this functional interaction between SWI/SNF and RB. They first demonstrated the interaction with a yeast two-hybrid system and showed that RB bound specifically to BRG1 and BRM and that the RB-dependent growth inhibition was dependent on the binding of RB to these SWI/SNF subunits (39). Next, they showed that the re-introduction of BRG1 or BRM caused growth inhibition that was dependent on members of the RB family, including RB, RB2 (p130), and to a lesser degree, p107 (8). This clearly established the functional interaction and RB dependence on the SWI/SNF complex. Several labs thereafter demonstrated that the growth inhibition induced by the ectopic expression of p16 or by the constitutively active isoform of RB required a functional SWI/SNF complex (9, 10, 40–42). Specifically, neither transduction with p16 nor transient transfection with RB could effectively induce growth arrest in BRG1/BRM-deficient cell lines (e.g., SW13, C33A, H522, etc.); at the same time, the re-expression of either BRG1 or BRM in conjunction with the re-expression of either p16 or constitutively active RB were both sufficient to restore RB-dependent growth inhibition. Interestingly, growth inhibition can also occur in the absence of RB in cell lines such as C33A and Saos-2, which harbor a functional p130 protein (43, 44). This is important because unlike RB, p130 is rarely mutated in cancer and retains its function in the control of the G1 to G0 transition, which is necessary for cancer stem cells to become quiescent (45). This fact coupled with the need for p130 to bind and cooperate with SWI/SNF might explain why *BRG1* and *BRM* are not frequently mutated. The epigenetic regulation of *BRG1* and *BRM* would allow cancer cells to become quiescent and thereby escape the cytotoxicity of standard chemotherapy as well as maintain their ability to subsequently re-enter the cell cycle. This mechanism could also allow cancer cells to delay re-entry into the cell cycle, which could explain why cancers can recur at various times after their initial discovery and treatment.

Interestingly, in most reviews and papers, SWI/SNF inactivation is infrequently considered as an alternative method of inactivating the RB pathway. Since the loss of BRG1, BRM, or both is commonly observed in many cancer types, the disruption of the RB pathway likely occurs from a loss of SWI/SNF activity in a subset of tumors (6, 19). Post-translational modifications via phosphorylation are also known to occur in both BRG1 and BRM (46). These modifications shift these proteins from the nucleus to the cytoplasm as cells enter M-phase, but this process is reversed when cells enter G1 phase (46). Whether cancer can highjack this regulatory mechanism as a means to inactivate either protein is not currently known. Some tumors, however, do display prominent cytoplasmic staining, which suggests that BRG1 or BRM must translocate to the cytoplasm (3, 5, 6). Further research is required to determine the scope of these epigenetic mechanisms and their implications for cancer development, progression, and recurrence.

## *BRM* Silencing and *BRM* Polymorphisms

The role of BRM in cancer initially appeared to be different from that of BRG1. In particular, a *BRM* knockout in animal models generates a phenotype that does not precipitate cancer *de novo*, but rather acts synergistically with carcinogens to yield tumors (3, 47). Hence, BRM loss would appear to foster cancer development when partnered with other changes such as exposure to carcinogens, rather than produce cancer by itself akin to driver oncogenes (48). In the pursuit to determine how *BRM* is silenced, it was found that in cell lines, *BRM* is epigenetically silenced, and its expression can be pharmacologically restored (49). It has since been found that the loss of BRM expression can be robustly reversed by flavonoids in general, and more specifically, by the synthetic flavonoid flavopiridol (50), which is now being tested in clinical trials (51). Recently, flavopiridol has been shown to inhibit the growth of rhabdoid tumors, which are deficient in BAF47 (SMARCB1; INI1) and commonly deficient in BRM as well (52). The induction of BRM is likely important to the growth-inhibitory mechanism of flavopiridol, as BRM silencing blunts flavopiridol-mediated growth inhibition in rhabdoid cell lines as well as in other cancer cell lines (50, 52). Interestingly, in addition to the ability of flavonoids to induce BRM in BRM-deficient rhabdoid tumor cell lines, the re-expression of BAF47 was also observed to have the modest effect on the restoration of BRM (52), indicating the existence of a cross-regulatory mechanism between SWI/SNF subunits. The re-expression of BAF47 has no effect, however, on HDAC9 or GATA3 expression, which are tightly linked with BRM silencing in cancer cell lines and primary tumors (21, 52). These data suggest that the restoration of BRM could be a mode of targeted therapy, since specific compounds (such as flavopiridol) are available that could be used, in theory, to inhibit cancer growth when applied to BRM-deficient cancers. The use of small molecule inhibitors or antibodies to block or inhibit aberrant activating kinases has become a standard clinical approach for the treatment of cancers. Yet, cancer is not only driven by aberrantly functioning gas pedals (oncogenes) but it also evolves as a result of the loss of brakes (tumor suppressors). Hence, the reversal of BRM silencing is another illustration of how an epigenetic mechanism can be reversed as a means for targeted therapy.

**Figure 1:**
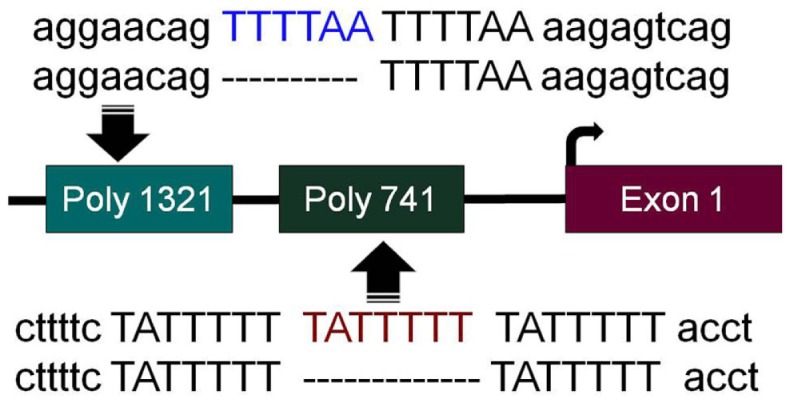
***BRM* promoter polymorphisms**. The two insertion polymorphisms located in the promoter of the *BRM* gene are illustrated here. The −1321 polymorphic site (poly 1321) (rs3832613 or rs5925917) is located 1321 bp upstream of the transcription start site and contains an additional 6 bp insertion, TTTTAA, to yield a duplicate repeated sequence “TTTTAA-TTTTAA,” while the wild-type allele only has a single “TTTTAA” sequence present. The −741 polymorphic site (poly 741) (rs34480940) is located 741 bp from the transcription start site and contains an additional 7 bp insertion, TATTTTT, to yield a triplicate “TATTTTT-TATTTTT-TATTTTT” sequence, while the wild-type sequence consists of the duplicate sequence “TATTTTT-TATTTTT”.

In order to successfully apply targeted therapies, ideally one should have biomarkers to identify those patients who would most likely benefit from a given therapy. To this end, during the pursuit to determine how *BRM* was silenced, we discovered two germline insertional polymorphisms within the *BRM* promoter (53). Both of these polymorphisms lie upstream of the transcription start sites within the first ~1300bp of the *BRM* promoter. The −741 polymorphic site is a triplicate repeat of the sequence TATTTTT, while the wild-type genotype is a duplicate repeat of this sequence (Figure 1). Similarly, the −1321 polymorphic site is a duplicate of the sequence TTTTAA, whereas the wild-type sequence contains this sequence only once (Figure 1) (53). Upon the discovery of these polymorphisms, two characteristics were readily observed. First, these two insertional polymorphisms have a remarkably high homology with MEF2 binding sites (54). In Caucasians, each of these polymorphisms is in Hardy–Weinberg equilibrium and occurs at a frequency of ~20% individually, while the frequency of the combined polymorphisms is roughly 6%. Second, the presence of either one or both polymorphisms statistically correlated with the loss of BRM expression in cancer cell lines (53). This statistical correlation was also found to be true in primary lung cancers (53). These polymorphisms would appear to be good surrogate biomarkers for *BRM* silencing in cancer given their strong correlation with BRM loss.

## Mechanism of the Epigenetic Silencing of *BRM*

As these polymorphisms were observed to be more prevalent in cancer cells that lack BRM expression, this suggested that they could be involved in *BRM* silencing. The high homology of these polymorphic sites with a known MEF2 binding site indicates that one of the MEF2 family members may be involved in *BRM* silencing. MEF2 transcription factors are known to silence genes by the recruitment of histone deacetylases (HDACs) (55, 56), and pan-HDAC inhibitors robustly restore BRM expression (57). Unfortunately, these pan-HDAC inhibitors also cause BRM acetylation and the subsequent inactivation of BRM. As such, these compounds cannot be practically used to restore BRM expression (16). This observation, however, indicated that HDACs must at least partially underlie *BRM* silencing. To this end, the shRNA-mediated knockdown of HDAC3 and HDAC9 robustly induced BRM, thereby showing that these specific HDACs are directly involved in *BRM* silencing (21). Similarly, the knockdown of both MEF2D and GATA3 also induced BRM, implying that these transcription factors are also part of the *BRM* silencing mechanism. While none of these proteins was found to be mutated in BRM-deficient cancer cell lines, HDAC9 and GATA3 were overexpressed in both lung cancer-derived cell lines and primary lung tumors, as well as in rhabdoid cell lines and primary tumors (21, 52). GATA3 and MEF2D, moreover, are known to bind to the HDAC9 promoter and drive HDAC9 overexpression (58). Just why HDAC9 overexpression appears to selectively occur in BRM-deficient cancer cells is not known. As HDACs are counter balanced by the activity of lysine acetyltransferases (KATs), it is not surprising that in BRM-deficient cell lines, ectopic expression of KAT6A, 6B, and/or 7 also induces BRM (21). Although KATs are often mutated or rearranged to form hybrid fusion proteins in many cancer types (32, 59), no alterations or mutations were observed in KAT6A, 6B, or 7 in BRM-deficient cancer cell lines (21).

Screening BRM-deficient cell lines with a library of kinase inhibitors led to the finding that the MAP kinase pathway also regulates *BRM* upstream of HDAC9 and GATA3 (52). Why an activated MAPK pathway leads to *BRM* silencing in only some circumstances is not known but appears to be linked to the presence of the *BRM* polymorphisms. The involvement of *BRM* polymorphisms has been further connected to *BRM* silencing by ChIP experiments showing that HDAC9 and MEF2D bind to the *BRM* promoter only when the BRM polymorphisms are present (52). Interestingly, MEF2D recruits HDAC9 to promoter regions, where it silences specific genes (56, 60). Hence, it appears that the activation of the MAP kinase pathway activates MEF2D, which in turn recruits HDAC9 to the *BRM* promoter by binding to the *BRM* polymorphisms. We surmise that this then leads to the removal of acetylated chromatin marks, such as H3K9 and H3K14 that control gene expression. The removal of these chromatin marks then prompts the closure of the chromatin and silencing of the *BRM* gene. Based on these data, the clinical targeting of *BRM* could be accomplished by inhibition of the MAP kinase pathway via FDA-approved inhibitors of B-raf such as vemurafenib and dabrafenib. Alternatively, as HDAC9 is a class 2 HDAC, its expression is relatively tissue-specific, and because it is highly overexpressed in BRM-deficient cancer cells, it might be a viable target for therapy. It is important to note that while *BRM* is silenced by this mechanism, it is unlikely that *BRM* is the only gene regulated by this aberrant gene silencing mechanism. Thus, targeting this mechanism may have broad anti-cancer effects, unrelated to *BRM*.

## *BRM* Polymorphisms, Cancer Risk and Clinical Outcome

While the loss of BRM *per se* does not independently drive cancer development in mice, BRM loss appears to tip the balance toward cancer development. The data from murine models indicate that *BRM* is not a tumor suppressor gene like RB or p53, but rather that it can function as a tumor susceptibility gene (3). Since the *BRM* polymorphisms appear to be surrogate markers for BRM loss (53), we surmised that these polymorphisms would correlate with cancer development. To investigate this possibility, a series of case control studies was undertaken, and each showed a statistical correlation with lung cancer risk with odds ratios between two and three (53, 61, 62). *BRM* polymorphisms have not only predicted lung cancer in repeated studies, but these markers also appear to be predictive of the cancer risk for other tumor types, including head/neck cancer (63). Loss of BRM protein expression in primary lung tumors has also been associated with a worse clinical outcome in patients (5, 6). *BRM* polymorphisms also have a statistical correlation with a worse outcome in patients with liver cancer (64). Based on these data, the *BRM* polymorphisms are emerging as predictive biomarkers in a variety of tumor types. Given the link between the *BRM* polymorphisms and BRM expression, it may be feasible to reverse the predictive trends of this biomarker by the induction of BRM or by the prevention of *BRM* silencing. To this end, flavonoids (42/42) from each of the six structural groups were observed to readily induce BRM, which suggests that BRM might play a part in the anti-cancer properties of flavonoids (50). It is intriguing to speculate that the role of *BRM* silencing in cancer risk and cancer development could be counter balanced by the dietary consumption of flavonoids, which readily restore BRM and foster the activation of RB (50).

## Mutations in SWI/SNF Subunits

Since *BRM* is reversibly epigenetically silenced in cell lines, it could be considered a target for therapy in tumors. For such therapies to be developed, however, it must be proven that *BRM* is not frequently silenced by mutations. As this axiom holds true for other SWI/SNF subunits, it is informative to determine the rate of a particular subunit’s mutation frequency versus its frequency of loss in a given tumor type. Mutations within the different SWI/SNF subunits have been well documented and described by a number of important reviews (65, 66). To ascertain the relative importance and role of each mechanism, it is useful to compare the frequency of mutations to other forms of gene alterations and inactivation. Four major sources have recently been mined to reveal such information about SWI/SNF: The Cancer Genome Atlas (TCGA) database, the Catalog of Somatic Mutation in Cancer (Cosmic) database, Sanger Sequencing publications, and available IHC data. In this review, we will focus on the comparison of the data in the Atlas database with published IHC data, and when possible, we will include mutation data from the Cosmic database. The Cosmic data will be denoted by percentage of mutations enclosed by brackets “(%)” and the TCGA data will be denoted by percentage of mutations enclosed by parentheses “(%).” In discussing mutations, it is prudent to acknowledge that mutations that change a single amino acid residue via missense might have a functional consequence, but this is not always easy to determine, and in most cases, functional data that explore the variable impact of such mutations are lacking. In contrast, gene alterations such as aberrant splicing, nonsense mutations (stop codons), or indels (frame shifts), which block the expression of a SWI/SNF subunit or result in the expression of a truncated protein, would be expected to have a much greater impact on the overall function of the mutated subunit, the SWI/SNF complex, and the pathways that are functionally dependent on this complex. NextGen sequencing studies have revealed a gamut of missense mutations that impact an unknown percentage of cancer cells within tumors, as summarized in a recent review (65). In general, current sequence data have not yet demonstrated specific hotspots in SWI/SNF subunits, making mutational analyses harder to interpret. Moreover, specific mutations or deletions in certain regions may fail to abrogate protein function. For example, the C-terminal truncation that causes the loss of the bromodomain of BRG1 has been reported *to not* block its ability to cooperate with RB and inhibit growth (10).

## Other Considerations in the Silencing of *BRG1* and *BRM*

**Figure 2:**
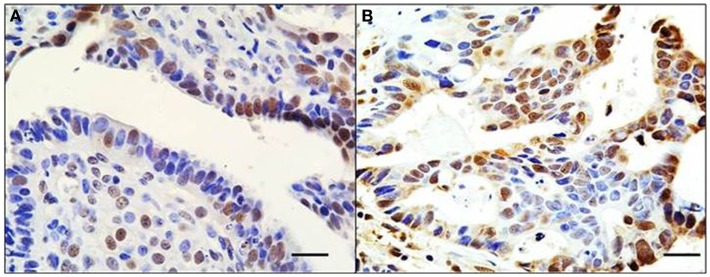
**Mosaic pattern of BRG1 expression in human tumors**. An ovarian **(A)** and liver **(B)** tumor both show an overtly mosaic pattern of BRG1 staining activity. BRG1-positive cells (brown) are observed adjacent to BRG1-negative cells (blue). This demonstrates the heterogeneous nature of these tumors with respect to loss of expression of the SWI/SNF subunits, in this case, BRG1. These pictures illustrate the problems in deciding whether a tumor is actually negative for expression of a given subunit. Original magnification: 63x; scale bar = 20 μm.

While it is frequently assumed that abrogating mutations such as indels and nonsense mutations (and probably missense mutations) are ubiquitous and homogeneous throughout tumors, immunohistochemical staining of tumors for BRG1 and BRM has shown that loss of expression has a variable penetrance in a given tumor (6). Such staining can vary by gene expression intensity and/or by the pattern of gene expression loss from a uniform to a mosaic or intermittent pattern as seen in Figure 2. In the absence of complete and homogeneous loss of gene expression, the impact of these other modes of gene expression alterations will invariably impact SWI/SNF and cancer to varying degrees. To complicate this matter, since SWI/SNF is a chromatin-remodeling complex, and thus a catalyst for opening and closing DNA to modulate gene expression, the level of expression of its subunits may not have a linear impact as is seen with other genes. Rather, there may be threshold effects where, despite a lower level of expression of a given subunit, SWI/SNF may have relatively preserved levels of function, and only when the subunit levels drop below a critical level does the SWI/SNF complex become functionally impaired. To this end, lower levels of BRG1 and BRM have been frequently observed in different tumor types (6). Therefore, the sub-threshold levels of a given subunit could be another mechanism by which SWI/SNF function might be perturbed. In support of this assertion is the observation that heterozygous loss of *BRG1* and *BAF47* predispose mice to tumor development (67–71). As many mutations in SWI/SNF subunits are missense, it is not known if these missense mutations function as dominant negatives or simply impair or abrogate function, causing a relative state of haploinsufficiency. In the setting where a given mutation (e.g., indel or nonsense) causes a loss of function of a given SWI/SNF subunit, these mutations usually only occur in one of the two alleles. The impact of the mutated allele depends in part on the status of the other allele. Such abrogating mutations in SWI/SNF subunits could have an impact, for example, if haploinsufficiency in a given subunit portends cancer development. A single allelic loss of *BAF47* or *BRG1* has been shown to promote a low but discernible rate of tumor formation in mice (71). Since excess SWI/SNF subunits are generally degraded (72), the protein expression from one allele could generate the required number of functional SWI/SNF complexes to avoid cancer development. If true, this mechanism could diminish the impact of allelic loss of a given subunit on the function of the complex and thus be considered a safeguard against transformation. However, when abrogating mutations (e.g., in *ARID1A*, *ARID1B*, and *PBRM1*) occur, they are frequently accompanied by loss of heterozygosity, leading to the complete loss of expression (see section below).

Another important consideration is the possibility of the functional redundancy of subunits, in particular BRM and BRG1, which are catalytic homologs within SWI/SNF (73, 74). While certain studies distinguish a functional difference between BRG1- and BRM-containing complexes, including differential interactions with specific transcription factors (75), other studies indicate that these subunits and their respective complexes can also substitute for one another if one subunit is suppressed or inactivated (2). This is most apparent in the studies of RB-mediated growth inhibition, where both BRG1 and BRM were shown to equally interact with RB and foster RB-mediated growth inhibition (8–10, 39). Given these findings, it is not surprising that in some tumors, such as lung cancer, the concomitant loss of BRG1 and BRM occurs in a significant portion (8–10%), compared with the loss of expression of either subunit alone, which occurs in 20–30% (5, 6, 76). Clearly, based on *in vitro* experiments, the value of inactivating both subunits eliminates any possibility of complementation and residual functionality of the SWI/SNF complex, in comparison with the silencing of either *BRG1* or *BRM* alone. In addition to BRG1 and BRM, functional redundancy may exist among other subunits (e.g., BAF60A, BAF60B, and BAF60C; or BAF155 and BAF170; or ARID1A and ARID1B), which suggests a mechanism by which the SWI/SNF complex may be further protected from cancer-driven subunit loss (2, 25).

## Loss of Heterozygosity and the SWI/SNF Subunits

The *BRG1* locus is known to be an area of loss of heterozygosity and lies adjacent to *LKB1* at 19q13.3 (77, 78). Similarly, the *BRM* locus is distal to p16 on the short arm of chromosome 9 at 9p23–24, which is another area of loss of heterozygosity (79–81). Both the *BRG1* and *BRM* loci are therefore areas where loss of heterozygosity is known to occur in primary tumors (2, 82). In many cell lines that lack BRG1 or BRM, one allele has been lost (unpublished data). *ARID1A* lies at 1q25.3, which is another area of loss of heterozygosity (83, 84). Interestingly, *ARID1B* is located at 6q25, an area of known loss of heterozygosity that has been linked to a documented genomic loss that drives inherited diseases of intellectual disability as well as multiple spinal meningiomas (85, 86). Moreover, the 6q25 locus is one of several regions that is frequently lost in central nervous system tumors, particularly in childhood neuroblastoma (87), where *ARID1B* and *ARID1A* are involved in 11% of these cases (88). *ARID2* maps to 12q12, an area that drives adenoid cystic carcinoma (89). *PBRM1* (*BAF180*) is located at 3p21.1, which is frequently lost in a variety of tumors (90). While 3p21 is frequently lost in lung cancer (91), *PBRM1* does not seem to be targeted in this cancer (92). In contrast, *PBRM1* involvement in 3p21 loss of heterozygosity in renal cell carcinoma is well documented and correlates with frequent mutations in *PBRM1* in this tumor (93). While loss of heterozygosity is frequently coupled to mutations that result in the inactivation of a target gene, loss of heterozygosity can also be coupled to epigenetic silencing. For example, most if not all BRM-deficient cell lines exhibit loss of heterozygosity (3, 52). Together, these observations regarding the loss of heterozygosity suggest that these genes can be more easily silenced when either mutational or non-mutational mechanisms occur in the remaining allele.

## Identification of BRG1 Mutations in Cell Lines Versus a Lack of Mutations in Primary Tumors

Defining *BRG1*-inactivating mechanisms in primary tumors has been hampered because of the initial discovery of *BRG1* mutations in cell lines (94, 95). A number of groups have sequenced *BRG1* in both BRG1-positive and BRG1-negative cell lines, and both missense and abrogating mutations have been reported in the vast majority of BRG1-deficient cell lines. In a minority of BRG1-deficient cell lines, such as C33A, H1573, and Panc-1, no detectible mutations have been found (unpublished data) (94). Interestingly, qPCR experiments by our lab have revealed that the levels of BRG1 mRNA in cell lines are much lower if *BRG1* is silenced by mutations compared to BRG1-deficient cell lines that lack mutations. We have also found that BRG1 is inducible in those cell lines that lack definable mutations (52). These findings give credence to the possibility that *BRG1* is epigenetically regulated in a subset of cancer cells. At least in cell lines, qPCR could serve as a preliminary screening mechanism to determine how *BRG1* might be silenced.

Some reports have implied that because *BRG1* is mutated in cell lines, it must also be mutated to the same degree in primary tumors (95), and BRG1 has been shown to be silenced by mutations in >65–70% of human cancer cell lines in a number of studies (94–96). These cell line data have driven speculation that mutations must be the primary mechanisms by which cancer targets and inactivates *BRG1*. Yet, the data from the NIH Atlas database demonstrate that the average weighted frequency (Table 1) and the average unweighted (Table 2A) frequency of abrogating mutations for *BRG1* in 23 [13] human tumor types is 0.4 and 0.21%, respectively, out of a total mutation rate of 3.75% (unweighted) and 2.41% (weighted)-which is well below the rate of *BRG1* silencing found in many human tumors (15–30%) according to published IHC data (5, 97). For example, according to the Atlas database, the rates of total mutations for *BRG1* in melanoma, pancreatic cancer, and head/neck cancer are 7.83, 7.69, and 5.88%, respectively, but the rate of abrogating mutations is <0.3, 1.1, and 0.33%, respectively (Table 2A); in comparison, the loss of BRG1 by IHC in these two tumors is far higher, ~10–27, ~10–25, and ~18%, respectively (98–100) (Marquez et.al. submitted Oncotarget 2015). Similarly, the rate of abrogating mutations of *BRG1* in colon and breast cancers is (0.0%) [1.28%] and (0.71%) [0.39%], respectively, while *BRG1* is silenced in >25% of these cancers as determined by IHC (Table 2A). In fact, the weighted average of abrogating and non-abrogating mutations as well as the total mutation rate for *BRG1* from the Atlas database from 23 tumor types is 0.21, 2.20, and 2.41%, respectively (Table 1). The data from the Atlas database are consistent with Sanger sequencing studies of *BRG1* in lung cancer, where abrogating BRG1 mutations (squamous and adenocarcinoma) are relatively low at 0.56 and 3.31% of tumors, while the loss of BRG1 expression occurred in ~15–30% (6, 19, 76, 101). Moreover, no abrogating mutations in *BRM* have been found in lung cancer. *BRM* is not significantly mutated (0.0%) as indicated by the Atlas data, which is clearly below the frequency at which its expression is lost in lung cancer (3, 5, 19).

**Table 1:** **Weighted averaged mutations of SWI/SNF subunits**.

	Total	Total	Total (%)
	Abr (%)	Non-Abr (%)	
SMARCD1	0.05	0.22	0.27
SMARCE1	0.10	0.27	0.40
SMARCD2	0.04	0.57	0.64
SMARCB1	0.13	0.43	0.66
SMARCD3	0.07	0.67	0.88
SMARCC1	0.06	0.91	0.97
SMARCC2	0.33	1.02	1.45
PBRM1	0.55	1.25	1.69
SMARCA2	0.09	1.84	1.96
SMARCA4	0.21	2.20	2.41
ARID1B	0.50	2.32	3.05
ARID2	1.68	2.40	4.24
ARID1A	2.55	2.16	4.83
Total	6.34	16.18	23.72
Adjusted total	5.19	10.30	15.49

*This table gives the percent abrogating, non-abrogating, and total mutations in each of the 23 tumor types for each SWI/SNF subunit shown. For each tumor type listed, the reported number of silent, missense, nonsense, and indel mutations as well as the number of deletions and splicing defects for each SWI/SNF gene were tabulated. Then, the total number of abrogating mutations (nonsense + indels + deletion + splicing defects) and the number of non-abrogating mutations (missense + silent) were calculated. The frequency for each cancer was calculated by recording the number of new cases for each of these 23 tumors then dividing by the number of total new cancer cases in United States. The weighted frequency was calculated by multiplying the frequency of abrogating, non-abrogating, and total mutations for each SWI/SNF subunit by the cancer frequency for each tumor type (Table S2 in Supplementary Material). The weighted mutation frequency for each SWI/SNF subunit was then calculated by summing the products of the frequency (cancer frequency times the mutation frequency) for each of the 23 cancer types. The adjusted total mutation frequency is the number of tumors with at least one SWI/SNF subunit mutation divided by the total tumor analyzed*.

**Table Notes**

Abrogating, non-abrogating, and total mutation rates from the TCGA and the Cosmic Databases for *SMARCA4* (*BRG1*): Table 2A, *SMARCA2* (*BRM*): Table 2A, *ARID1A* (*BAF250a*): Table 3A, *ARID1B* (*BAF250b*): Table 3B, *PBRM1* (*BAF180*): Table 4A, and *ARID2* (*BAF200*): Table 4B. A total of 23 tumors are listed in the first column. The second column lists the total number of tumors that were analyzed in TCGA database for each tumor type. Next, the percentages of non-abrogating (Nabr), abrogating (Abr) mutations, and total (Nabr + Abr) mutation for each tumor type are given. Similar data are then provided for the 13 tumors in the Cosmic database. The penultimate column lists the range of percent loss of *SMARCA4* according to immunohistochemical studies, and the final column provides the references for the IHC studies that were included. The final row gives the unweighted average percentage of Nabr, Abr, and total mutations for all 23 tumor types listed, for TCGA database and the 13 tumors listed for Cosmic database.

**Table 2A:** **SMARCA4 (BRG1)**.

Mutations	Tumor	TCGA: percentage			Tumor	Cosmic: percentage			IHC	References
	No.	Nabr (%)	Abr (%)	Total (%)		Nabr (%)	Abr (%)	Total (%)	Range (%)	
Acute myeloid leukemia	197	0.00	0.00	0.00						

Adrenocortical carcinoma	80	3.75	0.00	3.75						

Bladder urothelial carcinoma	237	5.49	0.42	5.91	Bladder	5.66	0.00	5.66	11.9	(3)

Brain lower grade glioma	289	3.11	0.00	3.11					19.2	(102)

Breast invasive carcinoma	981	0.71	0.71	1.43	Breast	0.78	0.39	1.17	30–40	Marquez et al. (submitted), (103)

Cervical cancer	39	2.56	0.00	2.56					22–40	Marquez et al. (submitted), (104)

Colon adenocarcinoma	269	0.00	0.00	0.00	Colon	5.75	1.28	7.03	26–48	

Endometrial cancer	248	9.68	0.00	9.68	Endometrium	9.61	0.36	9.96		

Esophageal cancer	282	2.10	1.10	3.20	Esophageal	4.62	0.60	5.23	11.9	Marquez et al. (submitted)

Glioblastoma multiforme	291	0.69	0.00	0.69					17–22	Marquez et al. (submitted), (105)

Head/neck cancer	306	5.56	0.33	5.88					18.0	

Kidney chromophobe	66	1.52	0.00	1.52						

Kidney renal clear-cell carcinoma	417	1.68	0.24	1.92	Kidney	1.05	0.00	1.05	70.0	Marquez et al. (submitted)

Kidney renal papillary cell carcinoma	112	5.36	0.00	5.36						

Lung adenocarcinoma	544	6.07	3.31	9.38	Lung	4.21	3.06	7.27	16–37	Marquez et al. (submitted), (19, 101, 106)

Lung squamous cell carcinoma	178	3.93	0.56	4.49					22–27	Marquez et al. (submitted), (5)

Ovarian carcinoma (serous)	230	1.74	0.00	1.74	Ovarian	1.61	0.00	1.61	18.7	Marquez et al. (submitted)

					Serous	1.20	0.10	1.30		

					Clear cell	9.10	0.00	9.10		

Pancreatic adenocarcinoma	57	6.59	1.10	7.69	Pancreatic	1.00	1.00	1.99	12–25	Marquez et al. (submitted), (98)

Prostate adenocarcinoma	251	0.00	0.00	0.00	Prostate	0.0	0.6	0.0	18–32	Marquez et al. (submitted), (106, 107)

Rectum adenocarcinoma	116	3.45	0.00	3.45						

Skin cutaneous melanoma	345	7.54	0.29	7.83	Melanoma	7.55	0.63	8.18	10–27	Marquez et al. (submitted), (100)

Stomach adenocarcinoma	245	3.27	0.82	4.08	Stomach	1.54	0.00	1.54	0–3	Marquez et al. (submitted), (108)

Thyroid carcinoma	405	0.99	0.00	0.99					7.9	Marquez et al. (submitted)

Uterine carcinosarcoma	114	0.88	0.88	1.75						

Hepatocellular carcinoma	202	6.44	0.99	7.43	Liver	1.63	0.61	2.24	40–60	Marquez et al. (submitted), (82)

Unweighted average		3.32	0.43	3.75		3.69	0.58	4.22		

A weighted frequency by cancer incidence shows that all mutations in SWI/SNF subunits occur in ~16% of these 23 human tumor types, where 15.5% of those tumors that harbor SWI/SNF mutations have more than one mutation. In comparison, the mutation rate of SWI/SNF subunits was reported as 20% in a recent review, which reported an unweighted average based on the number of tumors analyzed (65), rather than on the incidence and/or prevalence of these cancers; the latter more accurately reflects their true frequency in a population of cancer patients. In comparison, the mutational data on *BRM* in cell lines are consistent with experimental observed mutation rates in primary tumors; we did not observe any *BRM* mutations after we sequenced 20 primary lung cancers (3). Indeed, few if any cell line studies have shown that *BRM* is silenced by mechanisms other than epigenetic ones (3). These findings have been supported by the low levels of abrogating mutation rates (0.10%) [0.08%] detected when *BRM* has been sequenced in primary tumors, according to the Atlas database (Cosmic database) (Table 2B). This fact is critically important, as it shows that other mechanisms are at play that inactivate and silence *BRM*, and likely *BRG1*, in lung cancer. Given the available IHC data for BRM and BRG1 in other cancers, this observation also appears to be true in other cancer types, and similarly, other mechanisms likely underlie how *BRM* and *BRG1* are silenced in a variety of cancers.

**Table 2B:** **SMARCA2 (BRM)**.

Mutations	Tumor	TCGA: percentage			Tumor	Cosmic: percentage			IHC	References
	No.	Nabr (%)	Abr (%)	Total (%)		Nabr (%)	Abr (%)	Total (%)	Range (%)	
Acute myeloid leukemia	197	1.02	0.00	1.02						

Adrenocortical carcinoma	80	1.25	0.00	1.25						

Bladder urothelial carcinoma	237	4.64	0.00	4.64	Bladder	5.83	0.00	5.83	15.20	(3)

Brain lower grade glioma	289	0.00	0.00	0.00						

Breast invasive carcinoma	981	1.02	0.31	1.33	Breast	0.64	0.00	0.64	14.90	(3)

Cervical cancer	39	0.00	0.00	0.00						

Colon adenocarcinoma	269	0.00	0.00	0.00	Colon	1.31	0.16	1.48	70	(105)

Endometrial cancer	248	10.48	0.00	10.48	Endometrium	8.54	0.03	8.57		

Esophageal cancer	282	2	0	2.1	Esophageal	5.20	0.58	5.78	9	(3)

Glioblastoma multiforme	291	1.03	0.00	1.03						

Head/neck cancer	306	2.61	0.65	3.27					16	(63)

Kidney chromophobe	66	4.55	0.00	4.55						

Kidney renal clear-cell carcinoma	417	0.48	0.24	0.72	Kidney	0.88	0.00	0.88	53	Marquez et al. (submitted)

Kidney renal papillary cell carcinoma	112	0.89	0.89	1.79						

Lung adenocarcinoma	544	4.60	0.00	4.60	Lung	3.73	0.12	3.85	30–39	(3, 19, 101)

Lung squamous cell carcinoma	178	3.37	0.00	3.37					21–32	(3, 5)

Ovarian carcinoma (serous)	230	0.00	0.00	0.00	Ovarian (serous)	0.80	0.00	0.80	17–7	(3)

					Clear cell	0.00	0.00	0.00		

					Endometrioid	0.00	0.00	0.00		

Pancreatic adenocarcinoma	57	3.30	0.00	3.30	Pancreatic	1.23	0.01	1.23	50	(100)

Prostate adenocarcinoma	251	0.00	0.40	0.40	Prostate	1.00	0.00	1.00	35–47	(109, 110)

Rectum adenocarcinoma	116	2.59	0.00	2.59						

Skin cutaneous melanoma	345	4.64	0.00	4.64	Melanoma	1.25	0.00	1.26	20	(111)

Stomach adenocarcinoma	245	2.45	0.00	2.45	Stomach	0.00	0.00	0.00	42	(108)

Thyroid carcinoma	405	0.25	0.00	0.25						

Uterine carcinosarcoma	114	2.63	0.00	2.63						

Hepatocellular carcinoma	202	12.87	0.00	12.87	Liver	1.93	0.24	2.18	22.50	(82)

Unweighted average		10.59	0.10	11.09		2.16	0.08	2.23		

### Other mechanisms that silence *BRG1*?

A comparison of IHC data with NextGen sequencing data clearly shows that non-mutational mechanisms must also underlie most of the *BRG1* silencing in at least a subset of human cancers. Even before NextGen sequencing became a major experimental technique, Sanger sequencing in BRG1-negative tumors (as defined by IHC) showed that the frequency of abrogating mutations likely occurred in only a minority of these BRG1-negative tumors. For example, Oike et al. sequenced 16 BRG1-deficient lung cancers for *BRG1* mutations and found only one tumor with molecular alterations (missense mutation) (19). A limited analysis of gastric tumors also demonstrated a lack of *BRG1* mutations (112). Rodriguez-Nieto et al. analyzed 122 tumors, of which 46 (37%) were found to have low to null levels of BRG1; of these, only 5 missense but no abrogating mutations were found (97). Valdman et al. analyzed 21 prostate tumors by Sanger sequencing and found no detectable *BRG1* mutations (113), whereas BRG1 expression is lost in 18–32% of prostate tumors (Table 2A). Endo et al. analyzed 36 hepatocellular carcinomas, of which only four non-abrogating *BRG1* mutations were identified in two tumors (82). A combination of a loss of heterozygosity and mutations is a frequent mechanism by which genes are silenced in cancer, but despite the discovery of loss of heterozygosity at the *BRG1* locus, Gunduz et al. did not identify any mutations in *BRG1* in primary oral cancers (78). Yet BRG1 is lost in 18% of these tumors according to IHC data as illustrated in Table 2A.

In each of these cases, *BRG1* mutations occurred in a subset of tumors, but these observed mutational rates cannot explain why or how *BRG1* is silenced, given the higher rate of BRG1 expression loss. Hence, other mechanisms are likely occurring that silence *BRG1*. Whether silencing occurs by biallelic deletion of *BRG1*, aberrant BRG1 splicing, microRNA regulation, some other epigenetic mechanism or some combination of these mechanisms that has not yet been experimentally defined, is unknown. Indeed, there is clear evidence that each of these mechanisms can be involved in *BRG1* silencing. As noted above, both *BRM* and *BRG1* loci have been reported to be sites of loss of heterozygosity (76, 77, 79, 81). Given the frequent loss of one allele, it is possible that the second allele could also be lost, which could result in the silencing of these genes. In addition, microRNA (specifically miR-21), which is elevated in a variety of tumors and is functionally linked to cell growth and migration, has been reported to regulate BRG1 (114). A number of BRG1-deficient cell lines harbor aberrant BRG1 splicing defects where the BRG1 wild-type transcript is replaced by a transcript in which one or more BRG1 exons are omitted, which typically results in a frame-shifted protein. Epigenetic regulation of *BRG1* akin to the reported regulation of *BRM* has also been recently observed in BRG1-deficient cell lines. Understanding the mechanisms that drive *BRG1* silencing in primary tumors could be used to delineate which patients might benefit from *BRG1*-directed or other targeted therapies.

### *ARID1A*, *ARID1B*, *ARID2*, and *PBRM1* can be frequently mutated in specific tumor types

A series of NextGen sequencing studies have revealed that other SWI/SNF subunits are targeted by mutations. These studies have shown that *ARID1A* (*BAF250A*), *ARID1B* (*BAF250B*), *PBRM1* (*BAF180*), and *ARID2* (*BAF200*) have a weighted average mutation rate of 4.83, 3.05, 1.69, and 4.24%, respectively, in 23 tumors from the publicly available TCGA database (Table 1). In comparison, all the other SWI/SNF subunits have <2.4% weighted mutation rate. *ARID1A* is mutated in 7.3% of stomach, 11.9% of colon, 8.8% of pancreatic, 23.1% of cervical, 44.4% of endometrial, 18.1% of bladder, and 9.0% of adenocarcinoma lung cancers (TCGA database) (Table 3A). In those tumors where *ARID1A* is most frequently mutated, such as endometrial, bladder, pancreatic, and cervical cancers (66), more than ~60–80% of these mutations are abrogating and likely cause the disruption of protein expression. Similarly, the homolog of *ARID1A, ARID1B*, is frequently mutated in endometrial cancer (9.7%) and in hepatocellular carcinoma (10.4%) (Table 3B) (115), but the abrogation mutation rate is <1%, which indicates that these are missense (i.e., silent) mutations which do not effect ARID1B expression. In contrast, the abrogating mutation rate of *ARID1A* is 36.3% in endometrial cancer and 8.91% in hepatocellular cancer (Table 3A). Likewise, the rate of abrogating mutations in *PBRM1* is (21.1%) [22.5%] out of a total mutation rate of (26.6%) [27.5%] in renal cell carcinomas as indicated in the TCGA and Cosmic databases (Table 4A). For published studies where *N* > 220 cases, the mutational frequency for *PBRM1* in renal cell carcinomas has been reported to be 24, 38, and 41% by Kapur et al., Pena-Llopis et al., and Varela et al., respectively (116–118). In two of these studies, the delineation of mutation types was not given so the impact on expression could not be clarified. Nevertheless, the abrogating mutation rate can be estimated from the TCGA and Cosmic databases, where abrogating mutations are found to be 75–80% of the total mutations. Hence, applying this frequency of abrogating mutations indicates that the loss of PBRM1 occurs in about 20–33% of renal cell tumors. In the study by Pena-Llopis et al. (118), the abrogating mutational rate was 36%. In comparison, the loss of PBRM1 is between 53 and 70% from the published IHC data (119), which suggests that mutations are not the only mechanism underlying *PBRM1* silencing in renal cell carcinomas (119, 120).

**Table 3A:** **ARID1A (BAF250a)**.

Mutations	Tumor	TCGA: percentage			Tumor	Cosmic: percentage			IHC	References
	No.	Nabr (%)	Abr (%)	Total (%)		Nabr (%)	Abr (%)	Total (%)	Range (%)	
Acute myeloid leukemia	197	0.5	0.0	0.5						

Adrenocortical carcinoma	80	0.0	1.3	1.3						

Bladder urothelial carcinoma	237	5.9	12.2	18.1	Bladder	6.1	14.0	20.1	~34	(121)

Brain lower grade glioma	289	0.3	4.8	5.2						

Breast invasive carcinoma	981	1.2	2.0	3.3	Breast	1.8	1.7	3.5	60–65	(122–124)

Cervical cancer	39	5.1	17.9	23.1					6–20	(125, 126)

Colon adenocarcinoma	269	4.1	7.8	11.9	Colon	9.4	7.0	16.4	4–8	(127, 128)

Endometrial cancer	248	8.1	36.3	44.4	Endometrium	9.3	34.9	44.1	15–34	(129–131)

Esophageal cancer	282	2.80	3.50	6.40	Esophageal	3.5	13.3	16.8		

Glioblastoma multiforme	291	1.4	0.0	1.4						

Head/neck cancer	306	4.9	2.3	7.2					0.0	(128, 132)

Kidney chromophobe	66	0.0	0.0	0.0						

Kidney renal clear-cell carcinoma	417	1.0	0.5	1.4	Kidney	0.6	0.3	0.9	0–2	(128, 132, 133)

Kidney renal papillary cell carcinoma	112	4.5	0.0	4.5						

Lung adenocarcinoma	544	4.2	4.8	9.0	Lung	4.7	2.3	7.1	2.1–7.4	(128, 132)

Lung squamous cell carcinoma	178	6.2	2.8	9.0					10	(128)

Ovarian carcinoma (serous)	230	0.0	0.0	0.0	Ovarian	0.6	8.6	9.2	45–60	(134–140)

					Endometrioid	2.6	44.7	47.4	48–83	(135, 136, 138)

					Serous	0.1	1.0	1.1	60.0	(138)

					Clear-cell	1.7	60.9	62.6	41–75	(134–140)

Pancreatic adenocarcinoma	57	3.3	5.5	8.8	Pancreatic	1.5	5.7	7.2	6–8.5	(132)

Prostate adenocarcinoma	251	1.6	0.0	1.6	Prostate	1.2	0.3	1.6	0–5	(127, 128)

Rectum adenocarcinoma	116	1.7	2.6	4.3					1.0	(132)

Skin cutaneous melanoma	345	6.1	1.7	7.8	Melanoma	4.3	1.5	5.8		

Stomach adenocarcinoma	245	4.9	2.4	7.3	Stomach	4.2	12.6	16.7	14–25	(132, 141–143)

Thyroid carcinoma	405	0.0	0.0	0.0					10–14	Marquez et al. (submitted), (132)

Uterine carcinosarcoma	114	2.6	3.5	6.1					14.0	(132, 144)

Hepatocellular carcinoma	202	5.94	8.91	14.85	Liver	3.9	10.2	14.1		

Unweighted average		3.05	4.84	7.90		3.47	13.69	17.16		

**Table 3B:** **ARID1B (BAF250b)**.

Mutations	Tumor	TCGA: percentage			Tumor	Cosmic: percentage		
	No.	Nabr (%)	Abr (%)	Total (%)		Nabr (%)	Abr (%)	Total (%)
Acute myeloid leukemia	197	0.0	0.0	0.0				

Adrenocortical carcinoma	80	0.0	1.3	1.3				

Bladder urothelial carcinoma	237	1.3	2.1	3.4	Bladder	2.9	1.9	4.9

Brain lower grade glioma	289	0.7	1.4	2.1				

Breast invasive carcinoma	981	1.3	0.4	1.7	Breast	1.1	0.2	1.3

Cervical cancer	39	2.6	0.0	2.6				

Colon adenocarcinoma	269	5.6	0.7	6.3	Colon	4.6	0.9	5.5

Endometrial cancer	248	9.7	0.0	9.7	Endometrium	7.3	1.7	9.0

Esophageal cancer	282	2.50	0.70	3.20	Esophageal	1.2	0.6	1.7

Glioblastoma multiforme	291	1.0	0.0	1.0				

Head/neck cancer	306	5.9	0.7	6.5				

Kidney chromophobe	66	0.0	0.0	0.0				

Kidney renal clear-cell carcinoma	417	1.0	0.2	1.2	Kidney	1.7	0.0	1.7

Kidney renal papillary cell carcinoma	112	2.7	0.0	2.7				

Lung adenocarcinoma	544	5.0	0.6	5.5	Lung	4.3	0.6	4.9

Lung squamous cell carcinoma	178	6.2	0.6	6.7				

Ovarian carcinoma (serous)	230	0.0	0.0	0.0	Ovarian	0.2	0.4	0.6

					Serous	0.2	0.2	0.4

					Clear cell	0.0	12.5	12.5

Pancreatic adenocarcinoma	57	4.4	0.0	4.4	Pancreatic	1.2	0.1	1.3

Prostate adenocarcinoma	251	2.4	0.4	2.8	Prostate	1.3	0.3	1.6

Rectum adenocarcinoma	116	2.6	0.0	2.6				

Skin cutaneous melanoma	345	5.5	2.0	7.5	Melanoma	0.6	0.0	0.6

Stomach adenocarcinoma	245	4.5	0.4	4.9	Stomach	2.1	4.3	6.4

Thyroid carcinoma	405	0.2	0.2	0.5				

Uterine carcinosarcoma	114	0.9	0.0	0.9				

Hepatocellular carcinoma	202	9.41	0.99	10.40	Liver	1.7	0.2	1.9

Unweighted average		3.01	0.51	3.51		2.02	1.59	3.62

**Table 4A:** **PRBM1 (BAF180)**.

Mutations	Tumor	TCGA: percentage			Tumor	Cosmic: percentage			IHC	Reference
	No.	Nabr (%)	Abr (%)	Total (%)		Nabr (%)	Abr (%)	Total (%)	Range (%)	
Acute myeloid leukemia	197	0.00	0.00	0.00						

Adrenocortical carcinoma	80	1.25	0.00	1.25						

Bladder urothelial carcinoma	237	4.22	0.84	5.06	Bladder	5.8	0.0	5.8		

Brain lower grade glioma	289	1.38	1.04	2.42						

Breast invasive carcinoma	981	0.71	0.20	0.92	Breast	0.4	0.4	0.8		

Cervical cancer	39	5.13	0.00	5.13						

Colon adenocarcinoma	269	0.00	0.00	0.00	Colon	1.0	0.3	1.3		

Endometrial cancer	248	3.23	1.61	4.84	Endometrium	3.7	1.7	5.3		

Esophageal cancer	282	1.40	0.00	1.40	Esophageal	0.0	2.3	2.3		

Glioblastoma multiforme	291	0.69	0.00	0.69						

Head/neck cancer	306	2.29	1.31	3.59						

Kidney chromophobe	66	0.00	0.00	0.00						

Kidney renal clear-cell carcinoma	417	5.52	21.10	26.62	Kidney	5.0	22.5	27.5	54–68	(119)

Kidney renal papillary cell carcinoma	112	0.89	2.68	3.57						

Lung adenocarcinoma	544	1.65	0.55	2.21	Lung	2.2	0.5	2.6		

Lung squamous cell carcinoma	178	3.93	0.00	3.93						

Ovarian carcinoma (serous)	230	0.00	0.00	0.00	Ovarian (serous)	0.0	0.2	0.2		

					Clear cell	0.0	0.0	0.0		

					Endometrioid	0.0	0.0	0.0		

Pancreatic adenocarcinoma	57	2.20	1.10	3.30	Pancreatic	0.3	0.7	1.0		

Prostate adenocarcinoma	251	0.00	0.00	0.00	Prostate	0.0	0.0	0.0		

Rectum adenocarcinoma	116	3.45	0.86	4.31						

Skin cutaneous melanoma	345	3.19	2.61	5.80	Melanoma	0.6	0.0	0.6		

Stomach adenocarcinoma	245	2.86	0.82	3.67	Stomach	3.1	0.0	3.1		

Thyroid carcinoma	405	0.00	0.00	0.00						

Uterine carcinosarcoma	114	0.88	0.00	0.88						

Hepatocellular carcinoma	202	4.95	0.99	5.94	Liver	1.9	0.5	2.4		

Unweighted average		1.99	1.43	3.42		1.60	1.93	3.53		

Despite the apparent high frequency of *ARID1A* mutations from the TCGA database in some primary tumors such as pancreatic (8.8%), stomach (7.3%), and endometrial (44.4%), *BAF250* was initially noted to be infrequently silenced in cancer cell lines (96). *ARID2* was discovered by Weidong Wang in 2000 and found to be infrequently lost in cancer cell lines (145) but was recently reported to be mutated in 18% of melanoma and 7.9% of HCV-associated hepatocellular carcinomas (146–148). The TCGA database further shows that *ARID2* is also frequently mutated in (10.5%) of endometrial cancers (2.6%) of cervical cancers, and (5.1%) of bladder cancers (Table 4B). Similarly *PBRM1* was found to be infrequently silenced by mutations in human cancer cell lines (96); in contrast, *PBRM1* has been found to be silenced in 3.6–7% of stomach, endometrial, melanoma, pancreatic, cervical, bladder, and head/neck cancers based on the TCGA database. In comparison, PBRM1 is 3.8–7.4 times more frequently mutated in (26.62%) [27.5%] renal cell carcinomas than in these other tumor types.

**Table 4B:** **ARID2 (BAF200)**.

Mutations	Tumor	TCGA: percentage			Tumor	Cosmic: percentage		
	No.	Nabr (%)	Abr (%)	Total (%)		Nabr (%)	Abr (%)	Total (%)
Acute myeloid leukemia	197	0.0	0.5	0.5				

Adrenocortical carcinoma	80	0.0	0.0	0.0				

Bladder urothelial carcinoma	237	3.8	1.3	5.1	Bladder	3.9	0.0	3.9

Brain lower grade glioma	289	0.7	0.7	1.4				

Breast invasive carcinoma	981	1.1	0.3	1.4	Breast	0.6	0.5	1.2

Cervical cancer	39	2.6	0.0	2.6				

Colon adenocarcinoma	269	3.7	3.0	6.7	Colon	7.4	3.2	10.5

Endometrial cancer	248	8.1	2.4	10.5	Endometrium	8.9	1.8	10.7

Esophageal cancer	282	1.10	1.10	2.10	Esophageal	2.3	1.7	4.1

Glioblastoma multiforme	291	0.3	0.3	0.7				

Head/neck cancer	306	2.0	3.3	5.2				

Kidney chromophobe	66	0.0	0.0	0.0				

Kidney renal clear- cell carcinoma	417	0.5	0.2	0.7	Kidney	0.6	0.2	0.8

Kidney renal papillary cell carcinoma	112	0.9	0.9	1.8				

Lung adenocarcinoma	544	3.9	4.6	8.5	Lung	3.7	2.2	5.9

Lung squamous cell carcinoma	178	8.4	0.6	9.0				

Ovarian carcinoma (serous)	230	0.4	0.0	0.4	Ovarian (serous)	1.3	0.0	1.3

					Clear cell	0.0	0.0	0.0

					Endometroid	0.0	0.0	0.0

Pancreatic adenocarcinoma	57	4.4	0.0	4.4	Pancreatic	3.3	1.7	5.0

Prostate adenocarcinoma	251	0.8	1.2	2.0	Prostate	0.6	0.0	0.6

Rectum adenocarcinoma	116	5.2	1.7	6.9				

Skin cutaneous melanoma	345	9.6	8.4	18.0	Melanoma	4.1	6.1	10.2

Stomach adenocarcinoma	245	6.1	0.4	6.5	Stomach	3.1	0.0	3.1

Thyroid carcinoma	405	0.0	0.5	0.5				

Uterine carcinosarcoma	114	0.0	0.0	0.0				

Hepatocellular carcinoma	202	4.5	3.5	7.9	Liver	2.8	6.7	9.5

Unweighted average		2.72	1.39	4.11		2.85	1.61	4.46

To determine the role of mutations in the silencing of a given gene, one must compare the mutation rates and loss of expression data (IHC) for that gene. It should be noted that the validity of such an analysis is dependent on the accuracy of the reported mutational rates, which can vary depending on the study or database used. For this reason, we have chosen to standardize our comparison using the TCGA database. To this end, IHC data are primarily only available for ARID1A, BRG1, and BRM. The loss of ARID1A expression as determined by IHC and predicted by abrogating mutation rates as designated by “(%)” is illustrated in the following tumor types: ~14–25% (2.4%) of gastric cancers (141), 15–34% (36.3%) of endometrial carcinomas (129, 132, 149), 0–2% (0.5%) of renal cell cancers (121, 133), 60–65% (2.0%) of breast cancers (122–124, 150, 151), and 10% (2.8%) of squamous cell lung cancers (128). Hence, mutations do appear to account for the loss of ARID1A in certain tumor types, as the abrogating mutation rates are comparable to the loss of expression for endometrial tumors as determined by IHC. In other tumor types, however, the reported abrogating mutation rates are less than the frequency of gene expression. For example, nearly 60% of breast cancers demonstrate low to no ARID1A expression (123, 150), while 2.0% [1.7%] of breast cancers showed *ARID1A* mutations. Nearly 80% of these breast tumors demonstrated promoter methylation (122), and the heterozygous and homozygous allelic loss of *ARID1A* occurred in 35.2 and 2% of cases, respectively, in breast cancer (151). These data again show that mutations do occur in *ARID1A* and likely account for the loss of ARID1A expression in some tumor types, but that in other tumor types, other mechanisms of gene silencing must underlie *ARID1A* silencing. In addition, when mutations are detected in tumors, it does not necessarily mean that they are ubiquitously distributed in every tumor cell. As immunohistochemical studies accumulate, the mechanism of the silencing/abrogation of the other SWI/SNF subunits will be able to be evaluated.

*BRM* silencing is unlike *BRG1* silencing; with respect to *BRM*, mutations are rarely if ever documented in cancer cell lines (3). In fact, in the majority of BRM-deficient cell lines (20/20), BRM can be readily induced by a variety of agents (50). NextGen sequencing of BRM in numerous tumor types has substantiated this initial observation. The abrogating mutation and total mutation rate for BRM is (<1%) [<1%] and (<11.1%) [<2.6%], respectively, for each of the 23 tumor types available in the TCGA database and the 13 tumor types compiled from the Cosmic database (Table 2B). In comparison, the NextGen sequencing analysis of genetic disorders has revealed BRM germline mutations are higher in some genetic disorders than in cancer. For example, analysis of individuals with Nicolaides–Baraitser syndrome, a rare disease distinguished by sparse hair, unique facial morphology, distal limb abnormalities, and intellectual disability, showed that SMARCA2 (BRM) harbored missense mutations in 36 of 44 cases (~80%). Moreover, these mutations were harbored within conserved catalytic ATPase regions, suggesting that mutations do cluster within hotspots in certain diseases. Similarly, in another study, SMARCA2 (BRM) was mutated in 43% of patients with Coffin–Siris syndrome. Mutations in Coffin–Siris also occur in SMARCB1 (BAF47), SMARCA4 (BRG1), SMARCE1, ARID1A, and ARID1B, which attests to the role of the SWI/SNF complex in this genetic disorder.

## Genomic NextGen Sequencing Versus mRNA Splicing

Alternative splicing is a frequent mechanism that leads to aberrant or a complete loss of gene expression. As NextGen sequencing detects deviations in genomic DNA sequences within the coding regions of genes, splicing defects that occur by other mechanisms that do not involve the splicing junctions, but rather in acceptor or donor sites, may not be detected. Genomic DNA changes that affect the composition of branchpoint sequences or their relative position from the 3′ splice acceptor site of the downstream exon are also known to impact splicing, and certain missense mutations might be sufficient to create cryptic sites within the coding region. While non-mutational mechanisms can be inferred when the mutation rates do not match the frequency of gene silencing, other abrogating mechanisms such as aberrant splicing could contribute to this observed difference. Testing in cell lines permits the resolution of this type of discrepancy, but analyses in tumors are much more complicated because tumors are heterogeneous and multiple mechanisms may be active in a single tumor. Moreover, the mRNA levels that result from aberrant splicing are much lower than the mRNA level derived from the normal tissue within most tumor samples; this fact further complicates the detection and analysis of aberrant splicing in tumors compared with cell lines that lack these constraints. Hence, the notion that mutations and non-mutational mechanisms must be mutually exclusive is not true. It is more likely that both mechanisms are operating independently of each other and that the prevalence of each mechanism is driven by the selection force confronted by the tumor in its environment.

## Rhabdoid Tumors, Mutations and the SWI/SNF Complex

*SMARCB1* (*INI1* or *BAF47*) was the first gene of the SWI/SNF complex that was linked to cancer. The discovery of the role of this gene in the genesis of malignant Rhabdoid pediatric sarcomas was interesting. Mouse models have shown that the inactivation of the *BAF47* gene is highly tumorigenic. One might have thought, therefore, that this gene would be targeted and activated in a wide range of other cancer types, given its apparent degree of tumorigenicity. However, this is not the case, as the Atlas database shows that mutations in *SMARCB1* are relatively infrequent; the average weighted abrogating and total mutation rate is only 0.13% and 0.66%, respectively, in the TCGA database (Table 1). Several other SWI/SNF subunits are also infrequently mutated: *SMARCD1*, *SMARCD2*, *SMARCD3*, and *SMARCE1* have average weighted mutation rates of 0.27, 0.64, 0.88, and 0.40%, respectively, in the TCGA database. These subunits, like *SMARCB1*, are smaller subunits within the SWI/SNF complex (i.e., the molecular weight of each is under 60 kDa). In comparison, the weighted mutation rates of the large subunits that are in the 180–250 kDa range are generally higher than those of the small subunits, which suggests that this observed difference in the mutation rates may in part be due to the size differences of the subunits. If the mutation rate for each subunit is adjusted for the size difference by dividing the weighted mutation rates by the length/size in 100 kDa, then the size-adjusted-mutation rates for *SMARCD1*, *SMARCD2*, *SMARCD3*, and *SMARCE1* become (mutation percentage/100 kDa): 0.43, 1.01, 1.42, and 0.69%, respectively. In comparison, the mutation percentages per 100 kDa for *BRG1*, *BRM*, *ARID1A*, *ARID1B*, *ARID2*, and *PBRM1* are 1.27, 1.03, 1.93, 1.22, 2.12, and 0.93%, respectively. When corrected for size, most SWI/SNF subunits harbor 1% (±0.4%) mutations per 100 kDa, while the standardized mutational rates of *ARID1A* and *ARID2* are about twice this rate. In most tumors, the mutation rate of *SMARCB1* is <1.0%. In cervical carcinomas, squamous cell carcinomas, and adenocarcinomas of the lung, however, the mutation rates are 5.10, 1.7, and 2.8%, respectively (Table S1 in Supplementary Material). Most of these mutations do not affect the expression of *SMARCB1* as they are silent or missense mutations. For lung and cervical cancers, the rate of abrogating mutations is 0.6 and 0.0%, respectively. Thus, <1:200 of these tumors harbor a mutation in a *SMARCB1* allele.

## Abrogation of *SMARCB1* in Rhabdoid Tumors Led to the Discovery of SWI/SNF Involvement in Cancer

In 1990s, *SMARCB1* mutations and the subsequent inactivation of *SMARCB1* in rhabdoid tumors led to the realization that the SWI/SNF complex is often a target in cancer. Analyses of the various subunits were pursued based on these facts, and it was assumed that the SWI/SNF complex requires each of its subunits in order to be functional. Therefore, it was surmised by Bernard Weissman and others that the other SWI/SNF subunits must be involved in cancer akin to the *SMARCB1* gene (96). This premise led to the finding that *BRG1* and *BRM* are frequently silenced either alone or concomitantly in lung cancer (6). However, a recent analysis of rhabdoids has shown that BRG1 and BRM are also lost in many Rhabdoid tumors (52). BRM is silenced in 60–70% of primary rhabdoid tumors and in >90% of rhabdoid cell lines (52). Interestingly, the mechanism of silencing of *BRM* in rhabdoid tumors appears to be identical to that reported in lung cancer (52). Hence, *BRM* silencing appears to be conserved between these two very different cancer types. Similarly, *BRG1* is silenced in about 50% of primary rhabdoid tumors and about 75% of rhabdoid cell lines (unpublished data). In BRM-deficient rhabdoid cell lines and in a subset of BRG1-deficient rhabdoid cell lines, each of these genes is epigenetically silenced, and their expression can be restored (52). Remarkably, both genes can be readily restored by flavopiridol in a subset of lung and rhabdoid BRG1/BRM-deficient cancer cell lines (50, 52). As BRG1 and BRM complement the function of RB and RB2 in growth inhibition, their induction could be a necessary prerequisite for the clinical activity of flavopiridol. The epigenetic silencing of *BRM* and *BRG1* is in concert with the fact that there are few mutated genes in rhabdoid tumors. Hence, the study of the epigenetic mechanism behind *BRM* and *BRG1* silencing in rhabdoid tumors and potentially in other tumor types would seem to be important and a novel avenue for cancer research.

## In Summary

In recent reviews of the role of SWI/SNF in cancer, the majority of mutations discussed are missense and not abrogating mutations, yet overwhelming data show that SWI/SNF can be inactivated by both mutations and non-mutational silencing. These findings demonstrate that SWI/SNF function must be negatively impacted even more frequently than suggested by Kadoch et al. (65). The exact frequency of SWI/SNF impairment cannot yet be estimated from mutational analyses alone, but based on currently available data, SWI/SNF is impaired in at least 16% via mutations – and possibly >90% – of cancers, if one considers all mechanisms of inactivation.

## Conflict of Interest Statement

The authors declare that the research was conducted in the absence of any commercial or financial relationships that could be construed as a potential conflict of interest.

## Supplementary Material

The Supplementary Material for this article can be found online at http://www.frontiersin.org/Journal/10.3389/fonc.2014.00372/abstract
