# Supplementary material for: Beyond Mutations: Additional Mechanisms and Implications of SWI/SNF Complex Inactivation
Source: Front Oncol. 2015 Feb 27;4:372. doi: 10.3389/fonc.2014.00372 (PMC4343012; doi:10.3389/fonc.2014.00372)
Supplement: Supplementary file 1 [file Table_1.PDF]

Supp. Table 1: SMARCB1 (BAF47)	Tumor	TCGA: Percentage			Tumor	Cosmic: Percentage		
Mutations	No#	Nabr	Abr	Total		Nabr	Abr	Total
Acute Myeloid Leukemia	197	0.00%	0.00%	0.00%				
Adrenocortical Carcinoma	80	0.00%	0.00%	0.00%				
Bladder Urothelial Carcinoma	237	1.27%	0.00%	1.27%	Bladder	1.90%	0.00%	1.90%
Brain Lower Grade Glioma	289	0.35%	0.69%	1.04%				
Breast invasive carcinoma	981	0.20%	0.00%	0.20%	Breast	0.00%	0.00%	0.00%
Cervical Cancer	39	5.13%	0.00%	5.13%				
Colon adenocarcinoma	269	0.00%	0.00%	0.00%	Colon	2.80%	0.25%	3.05%
Endometrial Cancer	248	3.23%	0.40%	3.63%	Endometrium	2.84%	0.71%	3.55%
Esophageal Cancer	282	0.00%	0.00%	0.00%	Esophageal	0.96%	0.00%	0.96%
Glioblastoma multiforme	306	0.34%	0.00%	0.34%				
Head/Neck Cancer	202	0.98%	0.33%	1.31%				
Kidney Chromophobe	66	0.00%	0.00%	0.00%				
Kidney renal clear cell carcinoma	417	0.00%	0.00%	0.00%	Kidney	0.60%	0.24%	0.85%
Kidney renal papillary cell carcinoma	112	0.00%	0.00%	0.00%				
Lung adenocarcinoma	544	2.21%	0.55%	2.76%	Lung	0.46%	0.09%	0.55%
Lung squamous cell carcinoma	178	1.69%	0.00%	1.69%				
Ovarian Carcinoma (Serous)	230	0.00%	0.00%	0.00%	Ovarian (serous)	0.60%	0.00%	0.60%
					Clear cell	0.00%	0.00%	0.00%
					Endometrioid	0.00%	0.00%	0.00%
Pancreatic adenocarcinoma	57	0.00%	1.75%	1.75%	Pancreatic	0.50%	0.00%	0.50%
Prostate adenocarcinoma	251	0.80%	0.40%	1.20%	Prostate	0.41%	0.21%	0.62%
Rectum adenocarcinoma	116	0.86%	0.00%	0.86%				
Skin Cutaneous Melanoma	345	2.61%	0.00%	2.61%	Melanoma	1.80%	0.00%	1.80%
Stomach adenocarcinoma	245	0.00%	0.00%	0.00%	Stomach	0.90%	0.00%	0.90%
Thyroid carcinoma	405	0.00%	0.00%	0.00%				
Uterine Carcinosarcoma	114	0.00%	0.00%	0.00%				
Hepatocellular Carcinoma	202	1.49%	0.99%	2.48%	Liver	0.48%	0.00%	0.48%
Unweighted Average		0.85%	0.20%	1.05%		0.95%	0.10%	1.05%
