# Supplementary material for: Beyond Mutations: Additional Mechanisms and Implications of SWI/SNF Complex Inactivation
Source: Front Oncol. 2015 Feb 27;4:372. doi: 10.3389/fonc.2014.00372 (PMC4343012; doi:10.3389/fonc.2014.00372)
Supplement: Supplementary file 2 [file Table_2.PDF]

Tumor Type	No. Cases per Year	Tumor Group % Incidence	Histology % Incidence	Tumor type % Incidence
Adrenal	600			0.0360%
AML	52380			3.1449%
Bladder	74690			4.4844%
Breast	232670			13.9696%
Cervical	12340			0.7409%
Renal	63920	3.838%		
Chromo RC			5%	0.1919%
Clear Cell RC			70%	2.6865%
Papillary RC			13%	0.4989%
Colon	136830			8.2154%
Uterine	52630	3.160%		
Endometrial			80%	2.5279%
Carcinosarcoma			4%	0.1264%
Brain Cancer	22910			
GBM	3437			0.2063%
Glioma	18328			1.1004%
Head/neck	55070			3.3064%
Lung	224210	13.462%		
NSCLC-AC			40%	5.3847%
NSCLC-SCC			30%	4.0385%
SCLC			19%	2.5577%
Melanoma	76100			4.5691%
NHL	70800			4.2509%
Ovarian	21980			1.3197%
Pancreatic	46420			2.7871%
Prostate	233000			13.9895%
Stomach	22220			1.3341%
Thyroid	62980			3.7814%
Liver	33190			1.9927%
Other	148835			8.9361%
Total	1665540			

Supplementary Table 2. Tumor Types According to Incidence. A total of 29 tumor types (first column) is listed along with the estimated number of cases of each tumor type per year in the United States (second column). For renal, uterine and lung cancers, the percent incidence for each subgroup is given in the third column. The percent incidence according to histological subtype of renal, uterine and lung cancers is given in the fourth column. Finally, the fifth column shows the percent incidence for each tumor type. AML: acute myeloid leukemia; chromo RC: chromophobe renal cell cancer; Clear cell RC: clear cell renal cell carcinoma; Papillary RC: Papillary renal cell carcinoma; GBM: glioblastoma multiforme; NSCLC: non-small cell lung cancer; AC: adenocarcinoma; SCC: squamous cell carcinoma; SCLC: small cell lung cancer; NHL: non-Hodgkin’s Lymphoma.
